# Criterion Validation Testing of Clinical Metrology Instruments for Measuring Degenerative Joint Disease Associated Mobility Impairment in Cats

**DOI:** 10.1371/journal.pone.0131839

**Published:** 2015-07-10

**Authors:** Margaret E. Gruen, Emily H. Griffith, Andrea E. Thomson, Wendy Simpson, B. Duncan X. Lascelles

**Affiliations:** 1 Comparative Pain Research Laboratory, Department of Clinical Sciences, College of Veterinary Medicine, North Carolina State University, Raleigh, North Carolina, United States of America; 2 Department of Statistics, North Carolina State University, Raleigh, North Carolina, United States of America; 3 Morrisville Cat Hospital, Morrisville, North Carolina, United States of America; 4 Center for Comparative Medicine and Translational Research, Department of Clinical Sciences, College of Veterinary Medicine, North Carolina State University, Raleigh, North Carolina, United States of America; 5 Center for Pain Research and Innovation, University of North Carolina School of Dentistry, Chapel Hill, North Carolina, United States of America; Colorado State University, UNITED STATES

## Abstract

**Introduction:**

Degenerative joint disease and associated pain are common in cats, particularly in older cats. There is a need for treatment options, however evaluation of putative therapies is limited by a lack of suitable, validated outcome measures that can be used in the target population of client owned cats. The objectives of this study were to evaluate low-dose daily meloxicam for the treatment of pain associated with degenerative joint disease in cats, and further validate two clinical metrology instruments, the Feline Musculoskeletal Pain Index (FMPI) and the Client Specific Outcome Measures (CSOM).

**Methods:**

Sixty-six client owned cats with degenerative joint disease and owner-reported impairments in mobility were screened and enrolled into a double-masked, placebo-controlled, randomized clinical trial. Following a run-in baseline period, cats were given either placebo or meloxicam for 21 days, then in a masked washout, cats were all given placebo for 21 days. Subsequently, cats were given the opposite treatment, placebo or meloxicam, for 21 days. Cats wore activity monitors throughout the study, owners completed clinical metrology instruments following each period.

**Results:**

Activity counts were increased in cats during treatment with daily meloxicam (p<0.0001) compared to baseline. The FMPI results and activity count data offer concurrent validation for the FMPI, though the relationship between baseline activity counts and FMPI scores at baseline was poor (R^2^=0.034). The CSOM did not show responsiveness for improvement in this study, and the relationship between baseline activity counts and CSOM scores at baseline was similarly poor (R^2^=0.042).

**Conclusions:**

Refinements to the FMPI, including abbreviation of the instrument and scoring as percent of possible score are recommended. This study offered further validation of the FMPI as a clinical metrology instrument for use in detecting therapeutic efficacy in cats with degenerative joint disease.

## Introduction

Many adult and geriatric cats suffer from Degenerative Joint Disease (DJD) and associated chronic pain.[1, 2] In the United States, there is no approved medication for the long-term treatment of chronic pain in cats, despite the clear need for such a treatment. Several factors impair the ability to find useful treatment options for cats with DJD, including the lack of clinically-applicable, validated screening tools to identify cats with DJD-associated mobility impairment and outcome measures to evaluate efficacy of therapeutics.

Diagnosis of DJD has historically relied on some combination of veterinary examination, radiographic evidence, and owner input.[3–6] Previously, our group developed an instrument, the Feline Musculoskeletal Pain Index (FMPI),[7–9] to address this need. In the current study, we further investigated the function and use of the FMPI, including its ability to detect improvement using the non-steroidal anti-inflammatory, meloxicam, as a therapeutic intervention.

Meloxicam is used commonly in veterinary medicine for the treatment of DJD-associated chronic pain in dogs.[10–12] Meloxicam is currently approved in Europe for use in treating chronic pain in cats, but has not been approved for this use in the United States. There have been several suggestions from open label studies that lower doses of meloxicam than that approved in Europe are effective in the management of feline DJD-associated pain.[13, 14]

The purposes of the current study were two-fold: to evaluate the effectiveness of a ‘low dose’ of oral meloxicam to improve mobility in cats with DJD-associated chronic pain as measured by actimetry (a non-invasive method to monitor activity), and to further refine and validate the FMPI and other outcome measures. The additional outcome measures included a clinical metrology instrument we have termed the Client Specific Outcome Measure (CSOM),[15] fashioned after the Cincinnati Orthopedic Disability Index described for use in dogs,[16] and owner assessments of quality of life and temperament. Recently, we reported that owners may be better able to discriminate withdrawal of active medication than withdrawal of placebo, and that return of clinical signs following a treatment period might be a useful measure of efficacy.[17] This approach was also included in the data presented here.

We hypothesized that a low-dose of oral meloxicam given once daily would increase activity counts in cats as measured by actimetry and that activity counts would significantly decrease following withdrawal of meloxicam. Further, we hypothesized that the FMPI and the CSOM would respond to treatment, and/or withdrawal of treatment, and show criterion validity with the actimetry measures. Specifically, we tested the responsiveness (ability to detect the effect of an analgesic treatment and ability to detect the withdrawal of an analgesic) of the FMPI and CSOM in a double-masked, cross-over, placebo-controlled study and concurrently evaluated their criterion validity (whether the changes detected by the instrument correlated with an objective measure of altered mobility) by objectively measuring changes in activity counts in cats with DJD-associated pain.

## Materials and Methods

This study was approved by the Animal Care and Use Committee (Protocol # 11-102-O), at North Carolina State University College of Veterinary Medicine (NCSU-CVM), and written owner consent was granted in each case following verbal discussion of the study. The reporting of data follows the CONSORT guidelines.[18]

### Study Design

This was a randomized, stratified, double masked, placebo controlled, crossover clinical trial. Outcome measures included changes in owner ratings as well as changes in activity (as measured by actimetry) after having been treated with the active drug or placebo. Additionally, adverse events and clinical pathology were outcome measures.

### Animals

Potential study subjects were identified from clinic records by area primary care veterinarians, or were self-referred by owners that had seen advertisements for the clinical trial. Animals enrolled in the study were all client owned animals with naturally occurring chronic musculoskeletal disease.

### Inclusion and Exclusion Criteria

Cats were eligible to participate in the study if they had a qualifying degree of owner-noted mobility/activity impairment (described in the next section), evidence of pain during manipulation of at least two joints or spinal segments during veterinary orthopedic evaluation (described below under *Orthopedic evaluation*), and radiographic evidence of degenerative joint disease in at least two of the painful joints or spinal segments (described below under *Radiographic evaluation*). Cats also had to be greater than 1 year of age and weigh more than 1 kg (2.2 pounds). Predetermined exclusion criteria for all cats included the presence of suspected or diagnosed infectious diseases, symptomatic cardiac disease, immune-mediated disease, neoplasia, inflammatory bowel disease, urinary tract infection, hyperthyroidism, and diabetes mellitus. These conditions were ruled out by careful review of the medical records, owner history, physical examination, complete blood count (CBC), serum biochemistry panels, and urinalysis. Cats with chronic kidney disease (CKD) up to and including IRIS stage 2 [19] were eligible to enroll, provided the disease was stable as determined by repeated serum biochemistry panels and urinalyses. Cats with CKD of IRIS stage 3 or 4 were excluded from the study.

### Owner Evaluation of Mobility Impairment

During the screening interview, owners were asked to identify three activities that their cat showed impairment in performing. All interviews were carried out by either the study veterinarian (MG) or the study research technician (AT) who worked together to ensure uniform client interviews. Suggestions of types of activities were given to owners (e.g., running, jumping up to a specified height, moving up or down stairs) but owners were encouraged to generate activities specific to their cat. The investigator recommended that the activity be something that the owner felt the cat would be able to perform again if pain was controlled. These three items were then used to construct the Client Specific Outcome Measures (CSOM) assessment described below, and used for future assessments if the cats were successfully enrolled. Owners rated their cat’s ability to perform each activity on a Likert type scale ranging from 4 (No Problem) to 0 (Impossible) with intermediate values of 3 (Mild difficulty), 2 (Moderate difficulty), and 1 (Severe difficulty). Owner ratings were converted to numerical scores, and total CSOM score represented the sum of these three scores with a possible range of 0–12. In order to ensure that cats enrolled in the current study had moderate to severe activity impairments, as rated by their owners, cats were eligible for inclusion if they received an owner-rated score of ≤5 on Day 0. Owners completed the CSOM without knowledge of the cut-off for inclusion.

### Orthopedic Evaluation

Orthopedic evaluations were carried out as previously described,[9] and were performed by a single veterinarian (MG) following pre-study training.[1, 7] Briefly, every joint and axial skeletal segment (cervical, thoracic, lumbar, and lumbo-sacral) was palpated and manipulated to evaluate for signs of pain and instability. As described in [9], the pain response for each joint was graded on the following scale: 0 = no resentment; 1 = mild withdrawal, mild resistance to manipulation; 2 = moderate withdrawal, body tenses, may orient to site, may vocalize or increase vocalization; 3 = orients to site, forcible withdrawal from manipulation, may vocalize, hiss, or bite; and 4 = tries to escape or prevent manipulation, bites or hisses, marked guarding of site. A total pain score was calculated as the sum of all the individual appendicular joint and axial skeletal segment pain scores.

### Radiographic Evaluation

Cats meeting eligibility criteria for owner-noted mobility/activity impairment and pain on orthopedic evaluation were sedated using a standard protocol and orthogonal radiographs were taken of every joint and spinal segment. Radiographs were reviewed for the presence of degenerative joint disease as previously described [7] by a board-certified veterinary radiologist masked to the presence or location of pain.

### Randomization Method

The cats were randomly allocated to one of two treatment sequences (meloxicam followed by placebo [PDPP Group], OR, placebo followed by meloxicam [PPPD Group]), which were assigned to clinical cases according to predetermined randomization tables. The first period for all cats was placebo (P), followed by either meloxicam or placebo (D or P), a masked washout period (P), and a crossover period with either meloxicam or placebo (D or P). Randomization was stratified by owner-rated degree of impairment based on total CSOM score. Subjects were categorized as Low Impairment (Level 1) with total CSOM scores of 3, 4, or 5, and as High Impairment (Level 2) with total CSOM scores of 0, 1, or 2.

### Masking

NCSU-CVM Pharmacy personnel packaged meloxicam or placebo for administration to each cat. Placebo was identical to drug, minus the active ingredient, meloxicam. Volume of drug or placebo administered was based on the weight of the cat at D0 (enrollment). The volume of placebo was matched to volume of drug for each cat. Packaging for drug or placebo product was identical to prevent identification of treatment group by owners or investigators, and bottles were identified only by the days they were to be administered. Individual bottles for each period were delivered to the owner with both investigator and owner remaining masked to treatment group.

### Study Timeline

**Fig 1:**
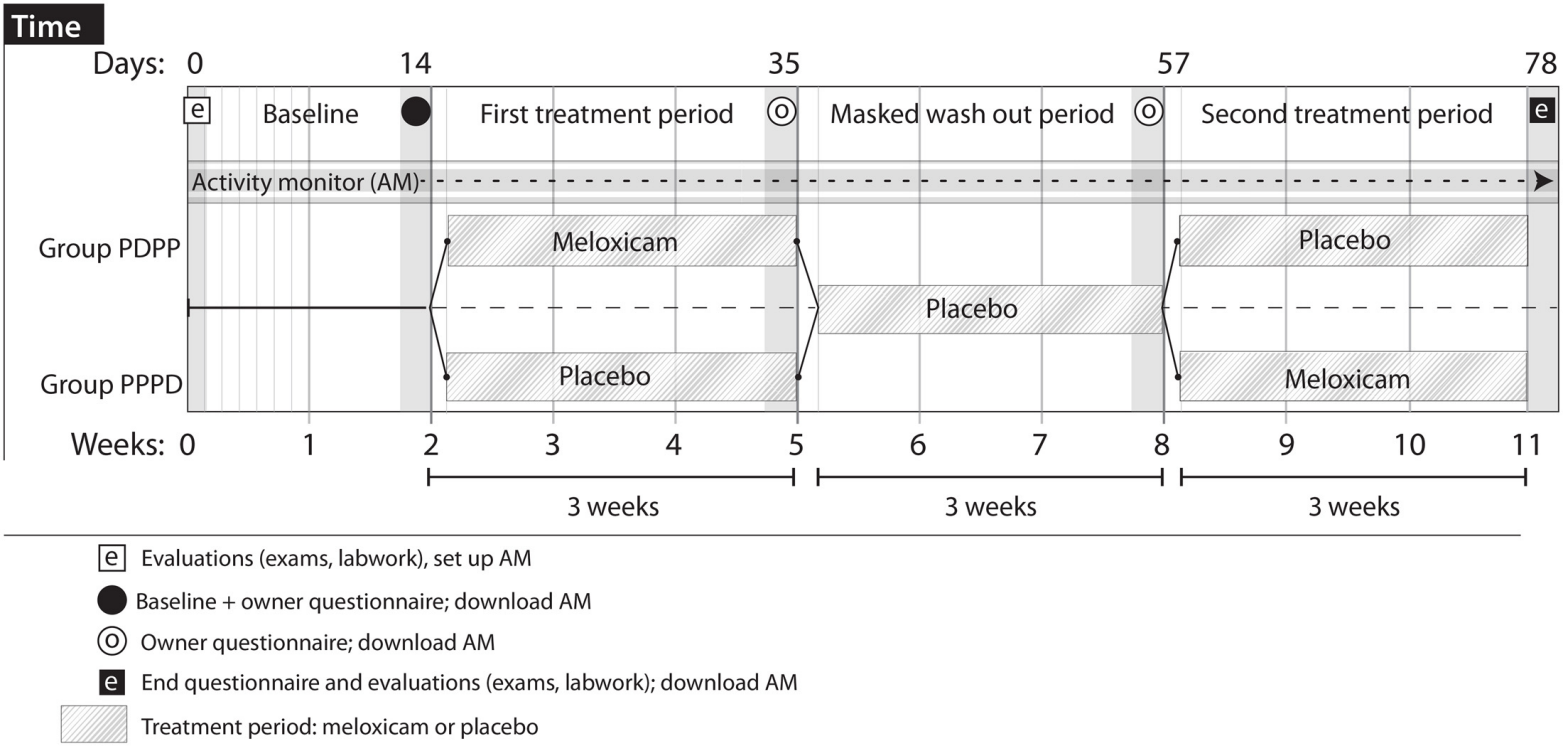
Study timeline and procedures completed at each timepoint.

Cats were screened on Day 0 with owner-interview, physical, orthopedic, and neurological examinations, radiographs, and labwork (CBC, serum biochemistry panel, urinalysis, and T4). Eligible cats were enrolled and fitted with an activity monitor (AM; Actical Z, Philips Respironics)^a^ worn for the duration of the study. Owners were given placebo (unmasked) at 0.07 mL/kg/day to be given orally during the baseline period (Days 1–14). Following Day 14, all future treatments were masked and volume-matched. Outcome measure questionnaires (described below) were completed at regular intervals (Fig 1). During treatment periods, cats received either placebo (0.07 ml/kg/day) or meloxicam (0.035 mg/kg/day). All cats received placebo (0.07 mL/kg) during the masked washout period. The study design was amended to add a second masked washout period (Days 78–99). As only 56% of enrolled cats completed this washout period, data from this period are not discussed here.

### Description of Outcome Measures

#### Safety and adverse events

Blood samples were taken from all the cats at Day 0 (baseline) and Day 78 to evaluate any changes in CBC or serum biochemistry values over the course of the study. Adverse events (AE) were defined as any observations in the cat that were unfavorable and unintended and that occurred during the study, whether or not it was considered to be treatment related. A serious adverse event (SAE) for this study was defined as any adverse event that was either fatal or life-threatening, required professional intervention (by a veterinarian) and considered by the investigators to be clinically serious or caused prolonged or permanent disability or disfigurement. Cats were observed throughout the study for AEs including but not limited to: changes in appetite or thirst, vomiting, diarrhea, changes in urination or defecation frequency and consistency, negative behavioral changes, neurological signs, lethargy or depression. Owners were asked to report any AEs at the time they occurred. Owners were given appropriate contact numbers for day or evening access to investigators in the case of any AEs or SAEs.

#### Efficacy outcomes

The primary outcome measures in the study were the AM output and the FMPI and CSOM scores. The secondary outcome measures were Change in QoL (QoLchange), Temperament and Happiness. These owner assessments were conducted at every time point (Days 0, 15, 36, 57, and 78).

#### Activity monitors

The AM and their use in cats have been previously described. [20–22] Activity monitors were mounted on a neck collar for all cats. Collars either belonged to the cats prior to the study, or were provided by the study for cats that did not have collars. Owners were given the option for a harness mounted activity monitor, but no owners selected this option. Activity monitors were set to collect data with an epoch length of 1 minute.

#### Owner assessments

**Feline Musculoskeletal Pain Index (FMPI)**: This questionnaire has been used in previous studies of cats with OA/DJD [8] and its development has been described.[7, 9] The version of the FMPI used in the present study differed from previous versions: the option to select ‘greater than normal’ had been deleted because previous analysis collapsed the ‘greater than normal’ and ‘normal’ category into one. The final two questions on the FMPI were converted from a Likert scale to a visual analog scale, and questions asking about ‘overall activity’, and overall quality of life were deleted following previous study results.[8] The FMPI queries owners on their cat’s ability to perform each of 17 activities (rated on a Likert scale from ‘Normal’ to ‘Not at all’) with an option to select ‘Don’t know or Not Applicable’. Two additional items (pain domain) ask owners to rate their cat’s level of pain on a standardized 100mm visual analog scale. Owner ratings were converted to scores ranging from 0–4 for each item with 0 = Not at all and 4 = Normal. Scores on the visual analog scale were calculated by measuring, in mm, from the start (zero point) to the owner’s mark, with 100 indicating ‘no pain’ and then dividing by 25 to align the scales for the entire instrument. The range of possible scores was 0–68 for items 1–17, and 0–78 for the full FMPI. On Day 0, owners completed a weighted FMPI (wFMPI), which asked owners to mark a visual analog scale to indicate the importance of each activity to their cat in addition to indicating their cat’s ability on the Likert scale. This importance scale ranged from 0 = No importance to 100 = extremely important. Importance scores for each item were calculated by measuring, in mm, from the start (zero point) to the owner’s mark. **Client Specific Outcome Measures (CSOM) Questionnaire**: As described above, the CSOM asks owners to identify 3 activities that their specific cat is impaired in performing, and to rate the cat’s level of difficulty in performing those activities on a Likert scale from ‘No problem’ to ‘Impossible’. For each time point after Day 0, owners were also asked to rate the change in their cat’s ability to perform the activity compared to before starting the medication for that period, and these ratings were transformed into numerical scores with Worse = -1, No Change = 0, and Improved = +1 for each activity. CSOM items were each presented in the same order for each cat at each visit. **Quality of Life and Temperament Questionnaire**: This assessment queried absolute Quality of Life and change in QoL, absolute Temperament and change in Temperament, and change in Happiness. Scores from the owner ratings were converted to numeric values as follows: QoL scores (QoL Scores) ranged from 1 = poor to 5 = excellent and a change in QoL (QoL Change) from -2 = much worse to +2 = greatly improved with 0 = no change in QoL; absolute temperament was a nominal scale, while change in temperament (Temp Change) ranged from -2 = much worse to +2 = greatly improved with 0 = no change in temperament. Happiness was rated only as change (Happiness Change), and was converted to a score ranging from -2 = much more unhappy to 2 = much more happy, with 0 = no change in happiness.

### Statistical Analysis

#### Handling of data

Safety was analyzed for all cats enrolled in the study using Day 0 and Day 78 labwork results and adverse events. Cats removed from the study due to AEs were included for safety using their initial labwork, and labwork obtained at the time of the AE. Cats removed from the study due to owner non-compliance were removed from the safety analysis if no follow-up labwork could be obtained. For all safety analyses, data collected on Day 0 were considered baseline. Comparisons to baseline refer to these data.

Efficacy was analyzed in both a parallel design (through Day 57) and a crossover design (through Day 78). Cats were removed from analysis of efficacy in the parallel design if they did not complete the study through Day 57, and from the crossover design if they did not complete the study through Day 78. For all efficacy analyses, data collected on Day 15 were considered to be baseline data. Stratification of the cats by CSOM score group (high impairment vs. low impairment) was incorporated into the randomization, but results were not analyzed separately for these strata. A p-value of 0.05 was considered significant unless otherwise noted.

#### Group demographics

Groups (PDPP group and PPPD group) were compared for distribution of age and weight using t-tests and gender of the cats using Chi-squared analysis. Groups were also compared on a number of environmental variables using Wilcoxon two-sample tests.

#### Safety

Paired t-tests were used to compare Day 0 laboratory results with end of study laboratory results (Day 78 for the majority of cases, earlier if cats were withdrawn from the study prior to Day 78). McNemar’s test was used to compare distributions on Day 0 and end of study lab work for categorical results. Wilcoxon signed rank test was used to compare Day 0 and end of study results for laboratory variables lacking a normal distribution (using a Kolmogorov-Smirnov test for normality). T-tests were also used to compare end of study results between cats in the PDPP group vs. those in the PPPD group (active medication would have ended 6 weeks prior versus 1 day prior to labwork, respectively in these groups).

#### Efficacy (parallel and crossover comparisons)

**Activity**: Data points (counts) for activity were generated at every minute of every day throughout the study. These activity counts were averaged for each 24-hour day to generate a single data point for each cat over each day and then the mean across a treatment period was calculated to create a single value for each cat for each treatment period. For between group comparisons, the group average activity counts for each day were used. These data were used for initial between group comparisons using t-tests. Due to marked inter-cat variability in activity, activity data were then summarized for each cat as a single value representing the average activity count per minute within a study period. Crossover analysis of improvement for both active treatment periods was performed using ANOVA. Paired t-tests of change in mean activity counts within each treatment group were used to compare within group changes during the active treatment period, and during the period following withdrawal of active treatment for the PDPP group. Paired t-tests were also used to compare between groups for improvement in mean activity count per cat per period by Day 36 over Day 15, and Day 78 over 57, as well as for decreases in activity counts by Day 57 over Day 36 between the PDPP and PPPD groups. **FMPI**: Data from the FMPI were summarized by cat as both raw score (total FMPI points for Q1-17, and Q1-19), and percent possible (%poss) score. Percent possible scores were used to adjust for the fact that some owners could not answer all items (for example, owners without stairs indicated a Not Applicable answer for items pertaining to stairs) and were calculated for items 1–17 and 1–19 by the following equations, respectively:

$$FMPI\%poss\;Score\;Q1-17=\left(\sum Q1-17\;scores\right)÷\left(number\;of\;questions\;answered\times 4\right)$$

$$FMPI\%poss\;Score\;Q1-19=\left(\left(\sum Q1-19\;scores\right)+\left(Q18÷25+Q19÷25\right)\right)÷\left(number\;of\;questions\;answered\times 4\right)$$

Repeatability (test-retest reliability) of the total score (for both 17 and 19 items) was evaluated in several ways. Spearman’s rank correlation coefficients between the Day 0 scores with the Day 15 (baseline) scores were calculated. Wilcoxon signed rank tests were used to check for systematic differences between D0 and D15 scores (if D15 scores were consistently lower or consistently higher than D0 scores). Bland-Altman plots were constructed to compare D0 and D15 scores.

For the parallel design analysis, t-tests were used to analyze improvement during treatment (raw scores and %poss scores on Day 36 minus scores on Day 15) as well as decline during the washout period (termed “deterioration” representing a return of clinical signs following withdrawal of previous medication; raw scores and %poss scores on Day 57 minus scores on Day 36). The FMPI was also analyzed in a success/failure paradigm and these results have been discussed elsewhere.[17]

For the crossover design analysis, improvement on Day 36 and 78 was analyzed using a general linear model. Both raw and %poss scores were evaluated.

In addition, in an effort to refine this instrument, following analysis of a treatment effect, we analyzed the owner-reported importance scores for each item, independence of items, and determined which items showed significant improvement and significant deterioration in the meloxicam treatment group during Days 15–36 and 57–78 respectively. Kruskal-Wallace test was used to compare FMPI items related to sociability with owner-rated temperament category. A Wilcoxon signed rank test was used to determine the items that showed significant improvement at Day 36 or deterioration at Day 57, and those items that discriminated between the PDPP sequence group and the PPPD sequence group at Day 36 and 57. **CSOM**: For the parallel analysis, total CSOM scores were analyzed using a Wilcoxon rank-sum test for Day 36 (improvement) and Day 57 (deterioration). For the crossover analysis, a general linear model was used to analyze improvement and deterioration in scores. Finally, a success-failure analysis was performed in which the number of cats in each group with a change in CSOM score of ≥2 for improvement at Day 36 over Day 15, and ≤ -2 for deterioration at Day 57 over Day 36 was compared using a Fisher’s exact test and previously reported.[17] **Quality of Life, Temperament, and Happiness**: For the parallel design analysis, Owner reported change in QoL, Temperament, and Happiness was dichotomized into improved/not (deteriorated/not) and analyzed using a generalized linear model. For analysis within the crossover design, dichotomized results were analyzed with a generalizing estimating equation (GEE).

## Results

### Enrollment and Distribution of Groups (Meloxicam-Placebo [PDPP] and Placebo-Meloxicam [PPPD] Groups)

A total of 66 cats were enrolled in the study. All cats had lived with their owners for at least 2 ½ months (range 2 ½ months– 16 ½ years; mean 10.25 years). Adequate randomization was achieved with no significant differences found between the groups for age, weight, or gender of the cats (Chi-square test, all ns) (Table 1). No significant differences were found for other demographic variables measured except for the variable “How long have you owned your cat,” where the median number of years for the PDPP group was 12, while the median number of years for the PPPD group was 10 (p = 0.02; Table 2). This difference, while statistically significant, was not considered clinically significant.

**Table 1:** Distribution of age, weight, and sex between treatment groups for all cats enrolled in the study.

	PDPP Group		PPPD Group		P-value (t-test)
Variable	N	Mean	N	Mean	
Age in years at start	33	12.6	33	12.5	0.93
Weight (kg)	33	5.58	33	5.63	0.90
	N	% Female	N	% Female	P-value (Chi-square)
Sex	33	48%	33	50%	0.90

**Table 2:** This table gives the results of Wilcoxon two-sample tests looking for differences between variables that were not controlled for in the treatment group selection.

	PDPP Group		PPPD Group			
Variable	N	Median	N	Median	Statistic	p-value
Number of people living in the house	33	2.0	33	2.0	1126.0	0.78
Number of children under the age of 10	33	0.0	33	0.0	1113.0	0.90
Number of people gone for more than four hours a day	32	1.5	32	1.0	1072.5	0.65
How long have you owned the cat? (years)	33	12.0	33	10.0	905.0	0.01
Number of pets in the house	33	4.0	33	3.0	1066.0	0.61
Number of cats in the house	33	3.0	33	3.0	1161.5	0.47
Number of dogs in the house	33	0.0	33	0.0	1037.5	0.31
Size of house (square feet)	33	2000.0	31	2400.0	1125.0	0.16
Percent of house access for cat	33	100.0	33	100.0	1050.0	0.43
Height easily jumped to (inches)	33	24.0	31	18.0	938.0	0.35

**Fig 2:**
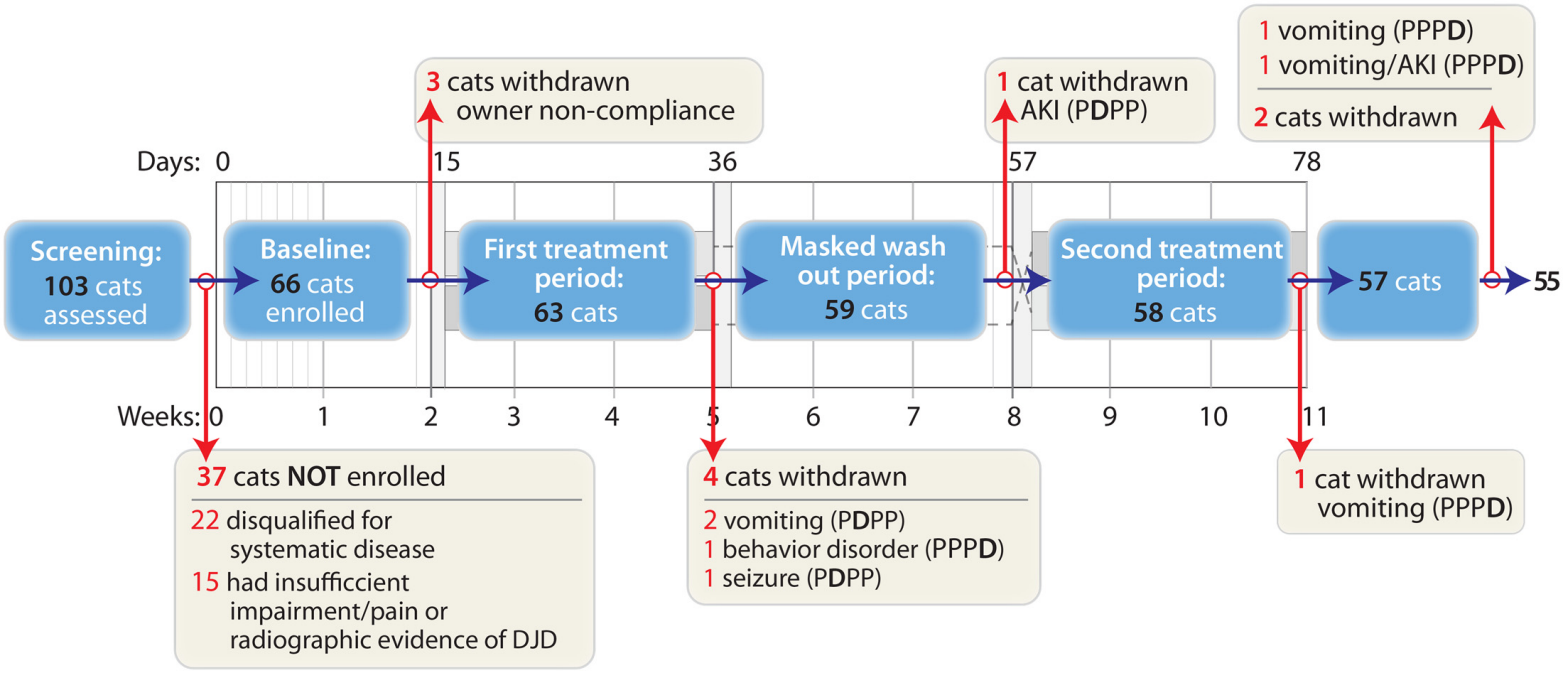
Participant flow diagram. Events occurring at each time point are outlined below. Cats removed from the efficacy analysis were retained for the safety analysis, unless otherwise noted. Cats are designated by the group they were part of (PDPP [meloxicam first] or PPPD [placebo first]).

Fig 2 outlines the flow of cats through the study following enrollment. In addition, one cat was removed partway through the study due to development/detection of clinically relevant hypertension with retinopathy (unrelated to study drug). Following stabilization and medical management, this cat was re-enrolled in the study under the same case ID number. Only data from the second enrollment were included in the analysis.

### Safety and Adverse Events

#### Labwork analysis

No significant changes were found between Day 0 and end of study laboratory results. When analyzed by final treatment (meloxicam or placebo), no significant effects seen except in albumin, where cats that were on meloxicam days 57–78 had a significantly higher change (decrease) in albumin from Day 0 to Day 78 (p = 0.009). There was not a statistically significant difference in albumin values between groups on either Day 0 (p = 0.971) or Day 78 (p = 0.084). Overall, two cats had albumin values below the normal range on Day 78, one was mildly decreased at the start of the study, while the other moved from the normal range to below normal (normal range 2.9 g/dL-4.0 g/dL).

#### Adverse events

All reported adverse events occurring in cats enrolled into the study are summarized in Table 3. Six cats were withdrawn from the efficacy analysis prior to Day 78 due to adverse events. Of these, four cats were withdrawn due to adverse events following meloxicam administration (three cats for vomiting and one for a single seizure), and two cats were withdrawn due to adverse events following placebo administration (one for behavioral disorder/hiding and one for acute kidney injury that was detected during the placebo period following meloxicam administration). Two additional cats had clinically relevant adverse events not reported or noted until the Day 78 exam. One cat had a mild increase in serum creatinine, while the other had been vomiting for several days prior to examination, and had a marked increase in serum creatinine requiring fluid therapy and hospitalization. These cats were withdrawn from the study with respect to efficacy analysis but are represented in the safety analysis.

**Table 3:** Summary of adverse events reported by owners during the course of the study.

Adverse Event	Placebo (number of cats with sign)/Number of cats removed	Meloxicam (number of cats with sign)/Number of cats removed	Cats removed from study prior to Day78 predominantly due to this AE
Gastrointestinal signs			
Vomiting	11/0	8/3	3
Diarrhea	2	2	0
Regurgitation	1	0	0
Flatulence	0	1	0
Loose stool	1	0	0
Constipation	1	0	0
Behavioral/Neurologic signs			
Head tilt*	1	1	0
Behavioral disorder not otherwise specified	2/1	1/0	1
Seizure	0/0	1/1	1
Urinary/Renal signs			
Nephritis	0	1	NA (end of study)
Acute Kidney Injury	1/1	1/0	1 (1 end of study)
Renal insufficiency	1	1	0
Urinary tract infection	2	0	0
Other Systemic Disorder			
Elevated ALT	0	1	NA (end of study)
Hypertension	0	1	0
Diabetes	1	0	NA (end of study)
Weight loss	1	0	0
Other labwork abnormalities	2	1	0
Other			
Skin lesion	0	2	0
Musculoskeletal disorder (owner concerned about antebrachial swelling)	1	0	0
Nasal discharge/sinus disorder	1	0	0
Aural discharge/infection	0	1	0

Listed are the number of cats with each sign while on placebo and while on meloxicam treatment. In addition, for any adverse event where cats were removed from the study prior to Day78 predominantly due to this AE, the number of cats removed from each group is listed.

* In one case (placebo), the head tilt was transient, in the other case (meloxicam), the head tilt was associated with severe otitis media.

### Efficacy

#### Actimetry

When data were averaged across cats, by day and treatment, significant differences between the treatment groups were found for Days 15–36 (first treatment period; t = 17.7, p<0.0001) Days 37–56 (t = 7.22, p<0.0001), and Days 56–78 (second treatment period; t = -2.35, p = 0.0235).

**Fig 3:**
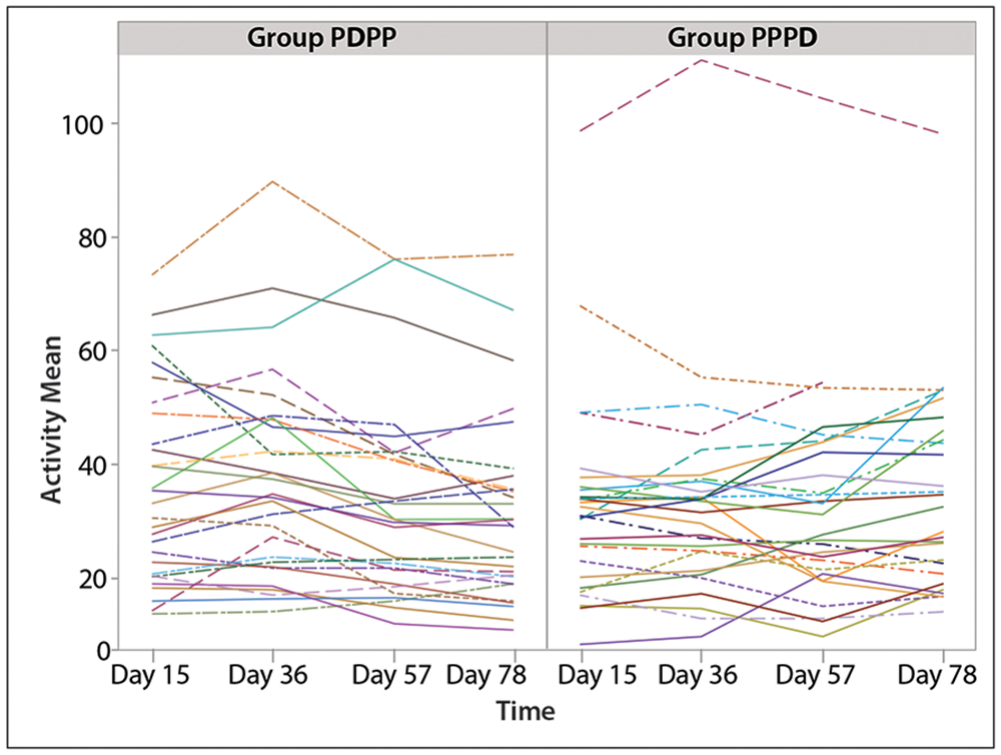
Plots of mean activity counts per day for individual cats in the PDPP (meloxicam first) and PPPD (placebo first) groups. These plots highlight the marked inter-cat variability in activity seen throughout the study.

Analysis of individual cat patterns of activity counts revealed marked inter-cat variability (Fig 3), suggesting that between group analyses may be less appropriate than within group analyses. The coefficient of variation between cats for the AM data during the baseline period was 50%. For remaining analyses, activity data were summarized as average counts per minute across each treatment period, with one value describing each cat for a given period. Using these data from the baseline period (Days 1–15) and the two treatment periods (Days 16–36 and Days 57–78), there was a strong treatment effect (p<0.0001, ANOVA) and a weak time and sequence effect (p = 0.088 and p = 0.081 respectively). Cats had increased activity counts when receiving meloxicam, and activity counts tended to be higher during the first treatment period than the second, and higher in the PDPP versus PPPD sequence cohort (Table 4).

**Table 4:** Summary of analysis of variance of average activity counts per minute per period and least-squares means from the crossover analysis.

Source	Numerator DF	Denominator DF	Type III Sum of Squares	F Value	Pr > F
**Treatment**	1	52	532.3	20.21	<0.0001
**Time**	1	52	79.5	3.02	0.088
**Sequence**	1	94.2	129.6	2.18	0.081
**Previous Activity**	1	52	48.2	1.83	0.182
**Variable**	**Level**	**Least Squares Mean**			
Treatment	Meloxicam	36.2			
	Placebo	31.8			
Time	Day 36	34.9			
	Day 78	33.2			
Sequence	PDPPP	35.6			
	PPPDP	32.4			

Averaging by cat across each period allows for a crossover analysis of improvement for both treatment periods.

Within group comparisons are summarized in Table 5, and show a non-significant increase in activity counts (p = 0.31) when meloxicam was administered, but a significant decrease in activity counts (p = 0.0008) when active medication is withdrawn in the PDPP cohort. The PPPD cohort showed a significant increase in activity counts when administered meloxicam (p = 0.0134), and no change from baseline to placebo, or placebo to placebo.

**Table 5:** Within group comparisons of activity data between periods.

**Group: PDPP**	**Mean Difference**	**Std. Dev.**	**T-statistic**	**P-value**
Days 15 to 36 –Days 1 to 15	1.3284	6.8628	1.04	0.3061
Days 37 to 56 –Days 15 to 36	-4.2448	6.1081	-3.74	0.0008
Days 57 to 78 –Days 37 to 56	-1.9101	4.9203	-2.09	0.0458
**Group: PPPD**				
Days 15 to 36 –Days 1 to 15	0.3497	4.8837	0.39	0.7027
Days 37 to 56 –Days 15 to 36	-0.3653	6.1219	-0.32	0.7503
Days 57 to 78 –Days 37 to 56	2.9776	5.9542	2.65	0.0134

Results from paired t-tests of change in mean activity counts for each cat within each treatment group, Days 1 to 15 versus Days 16 to 36, Days 37 to 56 versus Days 57 to 78. A positive result indicates higher activity counts during the later time period.

Comparing between groups for change (improvement) from baseline to the end of period 1, there was no difference between the cats receiving meloxicam in period 1 and those receiving placebo (p = 0.53), with both groups increasing in activity counts slightly (Table 6). However, cats that had received meloxicam during the first period showed a more significant decrease in activity counts during the masked washout (period 3) than the cats that had received placebo in the first period (p = 0.019).

**Table 6:** Between group differences in change in activity counts over the first treatment period and subsequent masked washout period.

	Group	Mean (SD)	t-statistic	p-value
Improvement (Day 36 –Day 15)	PDPP	1.33 (6.86)	0.63	0.534
	PPPD	0.35 (4.88)		
Deterioration (Day 57 –Day 36)	PDPP	-4.25 (6.11)	-2.42	0.019
	PPPD	-0.37 (6.12)		

Data are averaged across days by cat.

#### Feline Musculoskeletal Pain Index (FMPI)

**Fig 4:**
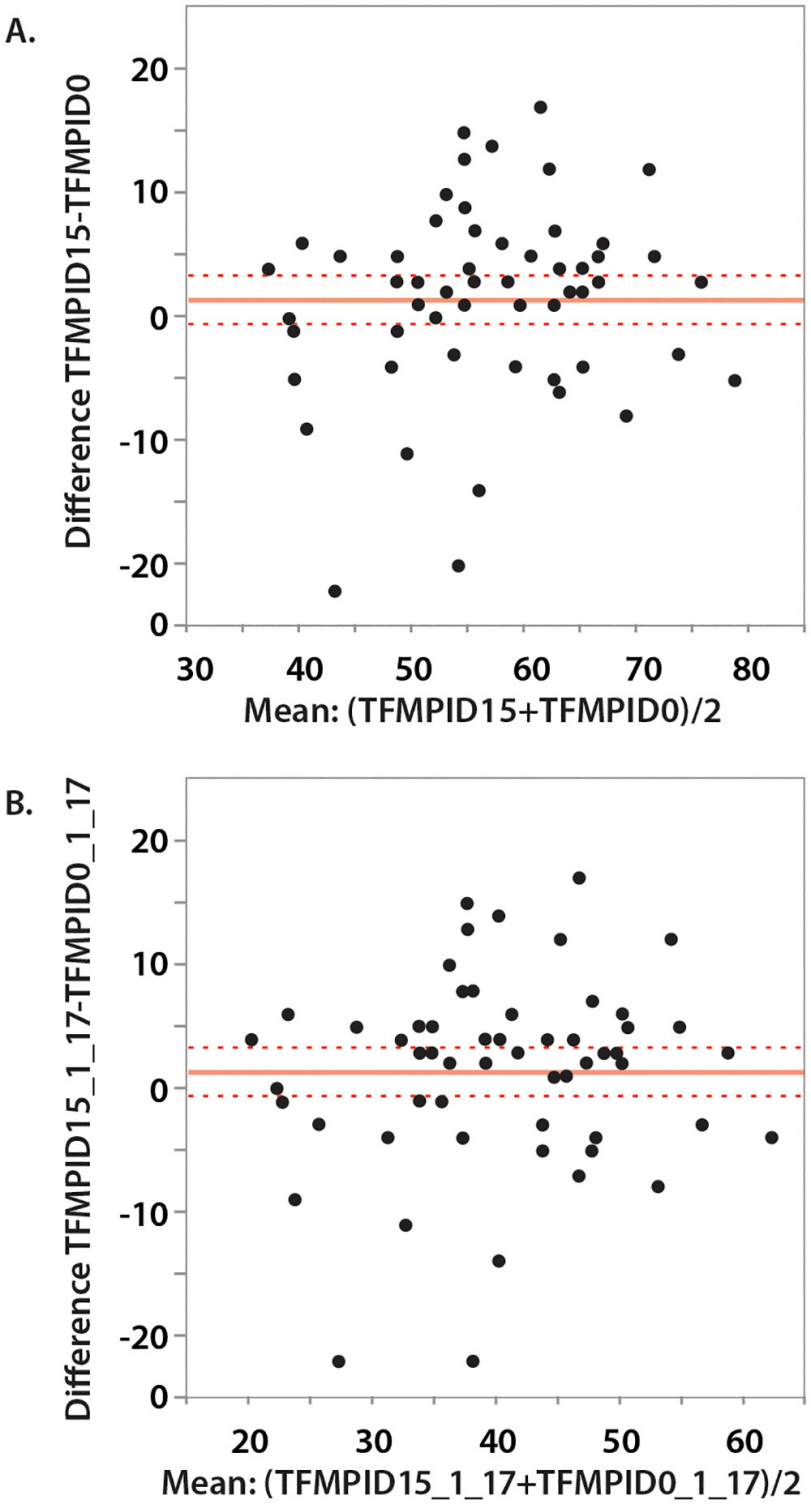
Bland-Altman plots of the FMPI for 17 items (A) and 19 items (B).

Correlations between D0 and D15 scores for the FMPI with 17 and 19 items were moderately strong (r_s_ = 0.72 and r_s_ = 0.73, respectively). The difference between the means was small (1.55 for the FMPI with 17 items, and 1.57 for the FMPI with 19 items), however Day 15 scores were significantly higher (indicating less impairment) than D0 scores for total FMPI with 17 items (S = 268.5, p = 0.031) and total FMPI with 19 items (S = 249.0, p = 0.041). Bland-Altman plots of the D0 and D15 data showed that there were 2 outliers, both cats belonged to same owner. The plots show the majority of cats were scored as being less impaired on D15 compared to D0. There was no obvious association between increasing score and an increase or decrease in scatter (Fig 4A and 4B). All points except the 2 outliers lay within +/- 2SDs of the difference.

Results for the parallel analysis are shown in Table 7. A significant difference (p = 0.048) in percent possible scores (for items 1–17) was found for deterioration, with the cats that had been on meloxicam during Days 15–36 showing a greater deterioration in %possible scores than the cats that had been on placebo. When raw scores (total of items 1–17 without accounting for percent possible) were compared, no differences between the groups were found for improvement at Day 36 or deterioration at Day 57.

**Table 7:** Summary of group comparisons of improvement / deterioration in the percent of possible points on the full FMPI questionnaire (Items 1–19), percent of possible points on Items 1–17 from the FMPI questionnaire, and improvement / deterioration in raw scores (Items 1–17) between Day 36 and Day 15 / between Day 36 and Day 57 respectively.

	PDPP		PPPD		T-test	
FMPI Q1-Q19: Percent Possible	Mean	Std.Dev.	Mean	Std.Dev.	T-statistic	p-value
Improvement, Day 36 –Day 15	0.107	0.106	0.100	0.100	0.26	0.794
Deterioration, Day 57 –Day 36	-0.056	0.121	-0.001	0.126	-1.69	0.097
FMPI Q1-Q17: Percent Possible						
Improvement, Day 36 –Day 15	0.102	0.105	0.092	0.101	0.38	0.706
Deterioration, Day 57 –Day 36	-0.055	0.113	0.006	0.118	-2.03	0.048
FMPI Q1-Q17: Raw Scores						
Improvement, Day 36 –Day 15	6.379	7.063	6.379	6.769	0.00	1.000
Deterioration, Day 57 –Day 36	-3.414	7.765	-0.276	7.695	-1.54	0.130

Both percent possible and raw scores were evaluated in a general linear model for improvement on Day 36 and 78 for the crossover analysis, results are shown in Table 8. Deterioration was not evaluated for the crossover as not all cats had a matched post-treatment washout period (Day 99). Percent possible was evaluated for both FMPI Q1-17 and FMPI Q1-19. A statistically significant treatment effect for improvement in percent of possible points was found for both 17 and 19 items (meloxicam treated cats had greater improvement than placebo treated cats), however the effect appeared to be stronger for the 17 items. Evaluation of raw scores for FMPI Q1-17 showed an improvement in meloxicam treated cats over placebo treated cats that was significant at the 10-percent level.

**Table 8:** Summary of general linear model analysis of improvement in the percent of possible points on the FMPI questionnaire (Items 1–19 and 1–17) and improvement in raw scores (Items 1–17) on Day 36 and Day 78.

Improvement (Day 36 and Day 78)			
FMPI Q1-Q19; Improvement in Percent Possible			
Source	DF	F Value	Pr > F
Treatment	1	4.72	0.034
Time	1	1.01	0.318
Sequence	1	0.00	0.952
Error	54		
Corrected Total	111		
FMPI Q1-Q17; Improvement in Percent Possible			
Source	DF	F Value	Pr > F
Treatment	1	6.07	0.017
Time	1	1.09	0.301
Sequence	1	0.02	0.882
Error	54		
Corrected Total	111		
FMPI Q1-Q17; Improvement in Raw Scores			
Source	DF	F Value	Pr > F
Treatment	1	3.30	0.075
Time	1	0.58	0.451
Sequence	1	0.04	0.852
Error	54		
Corrected Total	111		

**Fig 5:**
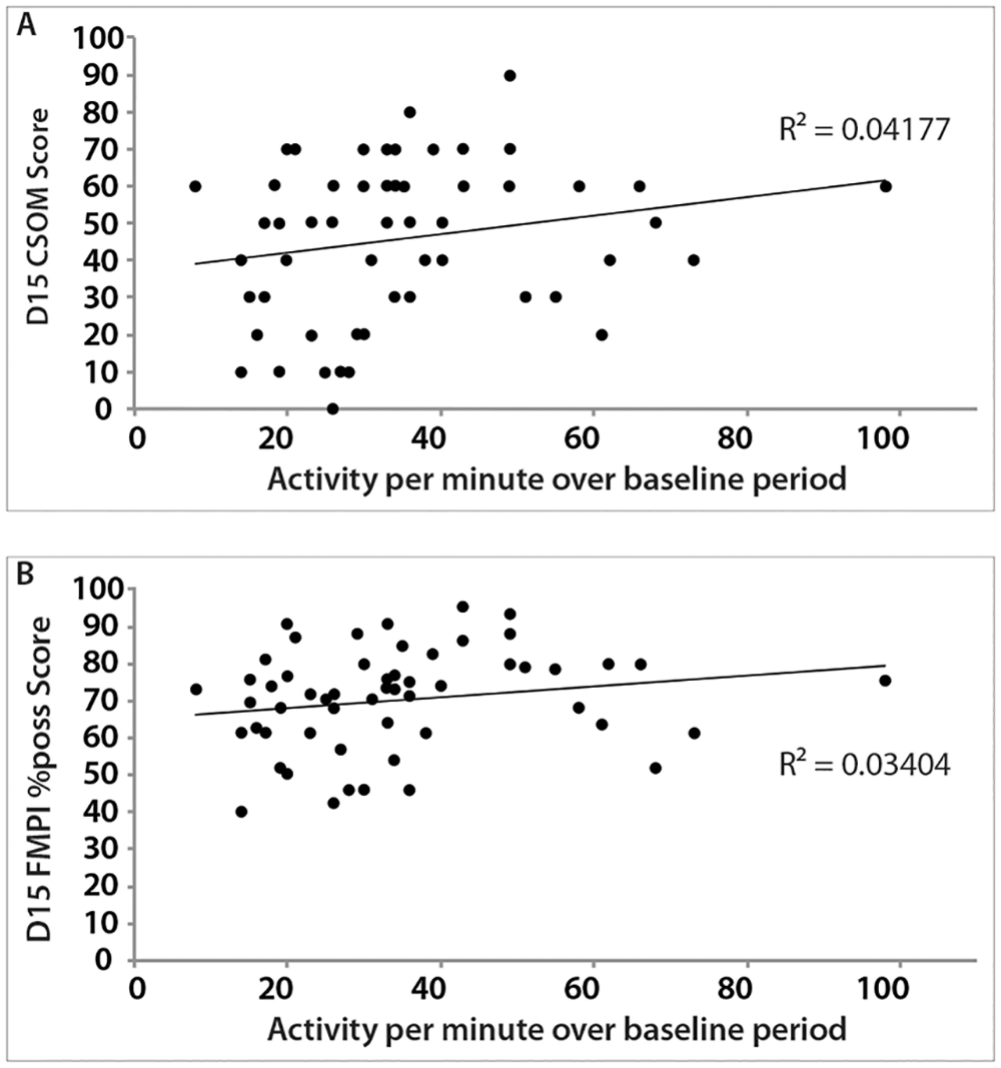
Relationship between average activity count and CSOM score at Day 15 (A) and FMPI %poss score at Day 15 (B).

There was no significant relationship between average activity count over the baseline period and Day 15 CSOM score (R^2^ = 0.042; P = 0.124) or Day 15 FMPI Score expressed as %possible (R^2^ = 0.034; P = 0.166) (Fig 5A and 5B).

Analysis of owner-rated importance scores showed high variability in responses. Importance scores relative to the overall median importance score across all respondents and all items indicated that items 1, 6, 7, 10, 11, 13, 14, 15, 16, and 17 were rated as relatively important to cats by owners of cats in the study. These items included questions about their cat’s ability to walk, go up and down stairs, and eat among others. Using these items alone did not detect improvement or deterioration. The items that detected significant improvement at Day 36 following meloxicam treatment were 3, 5, 6, 7, 8, 9, 10, 12, 15, 17, 18, and 19. Items that detected significant deterioration following meloxicam administration at Day 57 were items 9, 10, and 11. However, the only items that were able to discriminate between meloxicam and placebo at Day 36 (greater improvement in the meloxicam group) were items 1 (p = 0.019), 12 (p = 0.089), and 14 (p = 0.022), while those able to discriminate between withdrawal of meloxicam and withdrawal of placebo at Day 57 (greater deterioration in the cats that had moved from meloxicam to placebo) were items 10 (p = 0.018), 11 (p = 0.006), 12 (p = 0.030), and 16 (p = 0.060).

#### Client Specific Outcome Measures (CSOM)

For the parallel design, we first calculated Wilcoxon rank-sums tests for improvement of total CSOM scores on Days 36 (improvement) and 57 (deterioration). No statistically significant differences were found between the meloxicam group and the placebo group in this analysis. For the crossover design, we used a general linear model to analyze improvement in total CSOM scores and change in CSOM scores. In this model, there was a statistically significant time effect for change in total score (owners are more likely to report improvement at Day 36 than at Day 78) but no significant treatment effect (Table 9).

**Table 9:** Summary of general linear model analysis of improvement / deterioration in the total score across all three CSOM measures on Day 36 and Day 78 / on Day 57 and Day 99 respectively as well as change in total score.

	Source	D.F.	F-Value	P-value
Total Score, Day 36 and Day 78	Time (Day 36 and 78)	1	0.26	0.612
	Treatment (Meloxicam vs. Placebo)	1	0.62	0.433
	Sequence	1	0.37	0.544
Change in Total Score (Day 36 –Day 15, Day 78 –Day 57)	Time (Day 36 and 78)	1	23.09	<0.001
	Treatment (Meloxicam vs. Placebo)	1	0.06	0.814
	Sequence	1	0.03	0.862

Change was calculated as Day 36 –Day15, and Day 57 –Day 36.

#### Quality of Life, Temperament, and Happiness Questionnaire

For the parallel design, we looked at owner reported changes in QoL, Temperament, and Happiness at Day 36 (vs. Day 15) and Day 57 (vs. Day 36) for improvement from baseline, and deterioration from Day 36 levels, respectively. No statistically significant changes were found (Table 10). The deterioration in Happiness was significant at the 10-percent level, more cats that had received meloxicam during Days 15–36 had a decrease in Happiness at Day 57 than the cats that had received placebo during Days 15–36.

**Table 10:** Improvement in owner reported Change for Quality of Life, Temperament, and Happiness at Day 36 (from Day 15) and deterioration in owner reported Change for Quality of Life, Temperament, and Happiness at Day 57 (from Day 36).

Improvement at Day 36			Deterioration at Day 57		
Effect	Chi-Square	p-value	Effect	Chi-Square	p-value
Quality of Life	1.02	0.313	Quality of Life	1.27	0.259
Temperament	0.67	0.412	Temperament	0.42	0.518
Happiness	0.09	0.764	Happiness	2.98	0.084

When evaluating owner reported scores rather than owner reported change for QoL, there were no significant differences between groups for Day 36-Day 15 scores (improvement) or Day 57-Day 36 scores (deterioration).

For the crossover design, analysis of owner reported change in QoL, Temperament, and Happiness using a GEE analysis found a significant time effect for QoL (p = 0.017) such that owners were more likely to report improvement in QoL at Day 36 than at Day 78, regardless of treatment. No other significant effects were found for improvement.

## Discussion

With activity monitor counts used as our reference standard, we saw a significant treatment effect of low-dose oral meloxicam in increasing activity counts in cats with degenerative joint disease. Significantly higher levels of activity were detected in the meloxicam treated cats during the treatment periods. Inter-cat variability in activity counts were high. This is likely to be mainly due to differences in activity between cats, though reasons for these differences are unknown. Potential sources of variability include indoor vs. indoor/outdoor status, presence of stairs or size of home, and day of the week, as well as age, impairment, weight, and other factors. In this study, we restricted enrollment to cats that were indoors only, as indoor/outdoor cats have been shown to have different activity patterns, particularly if outside overnight.[23] This also decreases the effect that outside weather would have on activity. All cats in the study had approximately the same number of week and weekend days included in the analysis, and other factors were shown to be similar between groups of cats, so while these might contribute to individual variability, they were evenly distributed between treatment groups. In addition, recent data from our laboratory (Hansen, unpublished data) indicates that the output from different accelerometers can vary significantly (although each is stable over time), suggesting caution be exercised when comparing data between accelerometers. Considering both these aspects, we therefore consider within group comparisons more appropriate. When evaluating intra-cat/intra-group changes we found a significant treatment effect where meloxicam increased activity counts in cats with DJD. Further, we saw a significant deterioration in activity counts during the period following withdrawal of active medication in the PDPP sequence group. The same analysis was not available for all cats in the PPPD group. Other studies using meloxicam in cats with DJD or osteoarthritis have found similar treatment effects with meloxicam on improvement of activity counts.[8] Two studies using client-owned cats found that daily administration of meloxicam led to increased daily activity counts. In these studies, cats were selected based on owner-noted mobility impairment, and veterinary diagnosis of degenerative joint disease, and cats were maintained in their home environment throughout the study. Using laboratory housed cats, Guillot et al found that night time activity counts were higher in normal (non-arthritic) cats than in OA cats,[24] and later found that night-time activity counts increased in cats on meloxicam at 0.025 mg/kg and 0.05 mg/kg daily, though not in the cats receiving meloxicam at 0.04 mg/kg/day.[25]

The current study detected differences between cats receiving meloxicam vs. placebo using subjective measures of owner report captured with the FMPI. The FMPI was developed using appropriate methods.[7–9] In previous work, the FMPI was shown to have discriminatory validity,[7] and good repeatability (test-re-test reliability).[7] However, in the present study using cats with higher impairment, repeatability was not as good. In the present study, although test-retest reliability of the FMPI instrument was reasonable, scores on Day 15 were significantly higher than on Day 0. Although the actual difference in number of points was small, this indicates that owners are rating their cats as significantly less impaired on Day 15 following a 2-week period of known placebo administration. We believe this justifies the use of the Day 15 scores as the baseline. The period from Day 0-Day 15 may serve as a learning period for the owners as well as a chance for owners to better observe the behavior of their cats. By requesting owners to give a daily medication (in the exact same manner as in the masked portion of the study) we likely changed the observation of cats by owners, and perhaps also altered the behavior of the cat, which might explain some of the differences in repeatability found in the present study. The present study also included a new analysis, the percent possible calculation for the FMPI, as well as enrolling more cats than the previous evaluation of the FMPI.

As has been described for dogs with osteoarthritis [26], we also observed a sizeable caregiver placebo effect in owner reports during the first treatment period. Using the analysis of the percent possible score for the 17-item FMPI (excluding the two VAS scale items), this instrument is able to detect improvement in the crossover design, but not in the parallel design due to the caregiver placebo effect. This suggests that one way to adjust for the initial caregiver placebo effect is to use a crossover study design. Using the full cross-over design allowed for a difference between groups to be detected in objective actimetry during the second treatment phase. However, the full cross-over design creates a long, cumbersome clinical study. There are few masked, placebo controlled clinical studies assessing pain relief in client-owned cats with chronic pain [8, 15, 27] and large placebo effects have been seen in these studies as well. Although it has not been evaluated in detail, the placebo effect in these studies appears larger than is seen in comparable canine studies. This could be due to a lack of specificity in the CMI, or could be due to owners expectation of a positive effect combined with an overall lack of awareness of owners to their cat’s behaviors. Study design may also be improved by better training of owners to recognize signs of pain in their cats, particularly those that are most responsive to analgesics.

The use of a percent possible score for the 17-item FMPI was also able to detect a deterioration following withdrawal of active medication that is not seen with the withdrawal of placebo. This effect can also be seen with a success/failure paradigm for the FMPI and CSOM (though selection of threshold for the FMPI may require further evaluation).[17] These data parallel the decrease in activity counts seen when active medication is withdrawn. Interestingly, though only significant at the 10% level, owner ratings of cat happiness also show the deterioration effect following the masked washout. Our measures of temperament and quality of life, and change in temperament and quality of life did not detect the presumed efficacy of meloxicam, despite previous work indicating strong relationships between the presence of DJD and both temperament [3, 8, 28] and quality of life.[7, 29]

Return of clinical signs following randomized withdrawal of active medication is a clinical trial design that has been used in humans, however, to our knowledge, all studies using this approach fall into the category ‘Enriched Enrollment, Randomized Withdrawal’ (EERW).[30–32] EERW designs select for treatment responders at the start of study, followed by randomization to continuation of the treatment or a switch to a placebo or active control to measure efficacy. These studies may then compare the treatment to the placebo/active control. One complaint about this design is the length of time required for the study.[32] Our study design did not select for treatment responders, and we refer to our design using randomized allocation and withdrawal as the ‘RAW’ design. We believe RAW designed clinical trials may be very useful in evaluating analgesics for chronic pain management in cats, both for placebo controlled as well as analgesic comparison studies.[33] While replication is necessary, if effective, this study design provides for shorter study duration, and less cumbersome clinical trials.

Owner ratings of item importance for the FMPI were not fully indicative of the items in which improvement or deterioration were seen, or those where a difference was detected between meloxicam and placebo. Future work will investigate whether owner ratings of importance can be used to give weight to individual FMPI questions for individual owners, or used to refine the structure of the FMPI. One important consideration when considering refinements or modifications to the FMPI is the effect of the order of the questions, or whether particular questions are important in encouraging respondents to reflect on their cat’s behavior. The first step in selection of questions for an abbreviated FMPI would be to compare administration of the full instrument, with *a priori* decision to analyze only a subset of items, with a shortened form. Interestingly, while the FMPI, as percent possible, was able to detect a treatment effect, only one question, asking about the cat’s ability to stretch, distinguished between meloxicam and placebo in both the treatment and deterioration phase, and this was only if one accepted a p-value at the 10% level for the treatment phase. However, there was no significant relationship between baseline scores of ability to stretch and baseline activity counts, despite the fact that AM data was also able to detect treatment effects and the effects of treatment withdrawal. Previous studies with cats have looked at behavioral domains or specific behaviors that are responsive to analgesic treatment. A study by Clarke and Bennett showed owner ratings of improvement in willingness to jump, height of jump, and gait stiffness in cats with osteoarthritis that received meloxicam.[34] A second study by Bennett and Morton using meloxicam in cats with osteoarthritis found that owners rated positive changes in their cats in mobility, grooming habits, and temperament, with the greatest positive change in activity, though no placebo group was included for comparison.[28] A study by Klinck et al. used a survey to investigate, among others, additional signs for OA detection and monitoring in cats based on owners’ observations.[3] This study found that owners reported changes in several specific behaviors, with jumping, stair use, and alterations in gait being present in ≥75% of cats with OA. In addition, owners reported that jumping, stair use, speed, gait, mood, activity level, and daily schedule were perceived as responsive to treatment in ≥50% of the OA-affected cats. Future work should consider gathering video data on cats in the home environment, before and after treatment, and creating detailed ethograms of what behaviors are performed and change, as well as owner input on the changes they detected. This information may then form the basis of developing the next generation of CMIs.

Our data indicates that the incorporation of the final two questions on the FMPI (asking about pain levels over the last 3 weeks, and pain levels today) did not improve the sensitivity of the FMPI, and our current recommendations are that these are dropped from the instrument. We believe these changes can be made without further evaluation because they were the last 2 questions on the FMPI and so deleting them will not influence how earlier questions are answered.

Additional objective measures could also be incorporated into the management of cats with DJD, as well as for further development and validation of CMIs. Several objective measurement systems have been tested in cats, including peak vertical ground force reaction [24, 25, 35] and goniometry.[6] In particular, peak vertical ground force reaction (PVF) has been shown to be moderately feasible in client-owned cats (8/23 cats refused to traverse the walkway),[35] and able to distinguish between normal cats and those with DJD [24], but was not able to show a treatment effect for meloxicam in cats with DJD.[25] This measure was not included in the present study, however, it is possible that complementary measures to actimetry would allow for increased sensitivity in the refinement of CMIs.

Finally, we have suggested that the data from Day 99 might not be as useful as data gathered earlier in the study. There are several reasons for this observation, including the smaller number of cats completing this time point. We found clinician impressions that owners were experiencing study fatigue, and were not as diligent in completing their surveys during this final evaluation. In addition, to remain in line with the earlier study protocol, the cats themselves had their final appointments on Day 78, and labwork/physical exam findings had already been discussed. It is our feeling that the final masked washout period may have occurred after the study had almost “ended” in the minds of the owners, despite the investigators informing them that a further treatment period, which could be active medication or placebo, occurred during days 78–99.

In this study, we used the NSAID meloxicam as our therapeutic intervention. The low-dose of meloxicam used was generally tolerated well by most cats, and can be regarded as efficacious in the management of DJD-associated chronic pain. This study enrolled cats with mild-moderate renal insufficiency and, as reported in Gowan,[14] overall changes in creatinine and blood urea nitrogen were not observed. While the cats in the PPPD group had a higher decrease in albumin from Day 0 to Day 78 than those in the PDPP group, this is of unknown clinical significance in the absence of other changes. The number of cats showing vomiting were fairly evenly distributed to active and placebo treatments. However, rates of adverse renal effects including acute kidney injury were higher during or immediately following meloxicam administration than following placebo administration, warranting further investigation. In the most extreme case, a cat on meloxicam was vomiting for several days prior to the owner reporting this to investigators. It is not known, but is supposed, that earlier intervention would have mitigated the extent of this cat’s kidney injury, as meloxicam is tolerated fairly well by cats with chronic kidney disease that maintain euvolemia.[36] Given the therapeutic effectiveness of this medication, more sensitive early markers of injury, or screening tests able to predict tolerance of this class of drug would be of high value. This is especially important as recent work has shown that the overlap of DJD and CKD is great.[37]

### Conclusions

In conclusion, when evaluating treatments for DJD-associated chronic pain, we recommend a masked, parallel study design, which incorporates a masked washout period to allow for the assessment of deterioration (RAW design), and use of actimetry as a reference standard. We recommend the use of actimetry only for within group paired comparisons, and comparisons between groups in the degree of change. Actimetry values are too variable from cat to cat to allow for direct between group comparisons. Additionally, we recommend the use of the 17-item FMPI, expressed as a percent of possible, to detect deterioration. Finally, we recommend the use of success/failure criteria for the FMPI to detect deterioration, or the use of success/failure criteria for the CSOM to detect deterioration.

^a^ Actical, Philips Respironics, Bend, Oregon, USA

## Supporting Information

S1 Appendix Feline Musculoskeletal Pain Index. The FMPI is also available for download at: http://www.cvm.ncsu.edu/docs/cprl/fmpi.html as used for this study, and as the currently recommended 17-item version.
(PDF)
