# Supplementary material for: Criterion Validation Testing of Clinical Metrology Instruments for Measuring Degenerative Joint Disease Associated Mobility Impairment in Cats
Source: PLoS One. 2015 Jul 10;10(7):e0131839. doi: 10.1371/journal.pone.0131839 (PMC4498683; doi:10.1371/journal.pone.0131839)
Supplement: S1 Appendix — The FMPI is also available for download at: http://www.cvm.ncsu.edu/docs/cprl/fmpi.html as used for this study, and as the currently recommended 17-item version. (PDF) [file pone.0131839.s001.pdf]

Comparative Pain Research Laboratory
NC STATE UNIVERSITY

NAME: **DATE:**

FELINE MUSCULOSKELETAL PAIN INDEX

Please take some time to complete the following questions.

Please mark the circle that best describes your cat's ability to perform the following activities.

1. Walk and/or move easily?					
○	○	○	○	○	○
Normal	Not quite normal	Somewhat worse than normal	Barely, or with great effort	Not at all	Don't know or not applicable

2. Run?					
○	○	○	○	○	○
Normal	Not quite normal	Somewhat worse than normal	Barely, or with great effort	Not at all	Don't know or not applicable

3. Jump up (how well and how easily)?					
○	○	○	○	○	○
Normal	Not quite normal	Somewhat worse than normal	Barely, or with great effort	Not at all	Don't know or not applicable

4. Jump up to kitchen-counter height in one try?					
○	○	○	○	○	○
Normal	Not quite normal	Somewhat worse than normal	Barely, or with great effort	Not at all	Don't know or not applicable

NC STATE UNIVERSITY All material copyright of 'North Carolina State University' 2015 Version 9

Comparative Pain Research Laboratory
NC STATE UNIVERSITY

NAME: **DATE:**

Please rate your cat's ability to:

**5. Jump down (how well and how easily)?**					
○	○	○	○	○	○
Normal	Not quite normal	Somewhat worse than normal	Barely, or with great effort	Not at all	Don't know or not applicable

**6. Climb up stairs or steps?**					
○	○	○	○	○	○
Normal	Not quite normal	Somewhat worse than normal	Barely, or with great effort	Not at all	Don't know or not applicable

**7. Go down stairs or steps?**					
○	○	○	○	○	○
Normal	Not quite normal	Somewhat worse than normal	Barely, or with great effort	Not at all	Don't know or not applicable

**8. Play with toys and/or chase objects?**					
○	○	○	○	○	○
Normal	Not quite normal	Somewhat worse than normal	Barely, or with great effort	Not at all	Don't know or not applicable

**9. Play and interact with other pets?**					
○	○	○	○	○	○
Normal	Not quite normal	Somewhat worse than normal	Barely, or with great effort	Not at all	Don't know or not applicable

NC STATE UNIVERSITY All material copyright of 'North Carolina State University' 2015 Version 9

Comparative Pain Research Laboratory

NC STATE UNIVERSITY

NAME: **DATE:**

Please rate your cat's ability to:

**10. Get up from a resting position?**					
○	○	○	○	○	○
Normal	Not quite normal	Somewhat worse than normal	Barely, or with great effort	Not at all	Don't know or not applicable

**11. Lie and/or sit down?**					
○	○	○	○	○	○
Normal	Not quite normal	Somewhat worse than normal	Barely, or with great effort	Not at all	Don't know or not applicable

**12. Stretch?**					
○	○	○	○	○	○
Normal	Not quite normal	Somewhat worse than normal	Barely, or with great effort	Not at all	Don't know or not applicable

**13. Groom himself or herself?**					
○	○	○	○	○	○
Normal	Not quite normal	Somewhat worse than normal	Barely, or with great effort	Not at all	Don't know or not applicable

**14. Interact with you and family members?**					
○	○	○	○	○	○
Normal	Not quite normal	Somewhat worse than normal	Barely, or with great effort	Not at all	Don't know or not applicable

NC STATE UNIVERSITY All material copyright of 'North Carolina State University' 2015 Version 9

NAME: **DATE:**

Please rate your cat's ability to:

**15. Tolerate being touched and/or held?**					
○	○	○	○	○	○
Normal	Not quite normal	Somewhat worse than normal	Barely, or with great effort	Not at all	Don't know or not applicable

**16. Eat?**					
○	○	○	○	○	○
Normal	Not quite normal	Somewhat worse than normal	Barely, or with great effort	Not at all	Don't know or not applicable

**17. Use the litter box (get in and out, squat, cover waste?**					
○	○	○	○	○	○
Normal	Not quite normal	Somewhat worse than normal	Barely, or with great effort	Not at all	Don't know or not applicable

How does your cat feel?

18. Please mark the point on the line that best describes your cat's pain over the past two weeks:

No pain ______________________________ Severe pain ☐ Don't know

19. Please mark the point on the line that best describes your cat's pain <u>today</u>:

No pain ______________________________ Severe pain ☐ Don't know

NC STATE UNIVERSITY All material copyright of 'North Carolina State University' 2015 Version 9
